# Recent Progress on Epidemiology and Pathobiology of Porcine Circovirus 3

**DOI:** 10.3390/v13101944

**Published:** 2021-09-28

**Authors:** Si Chen, Liying Zhang, Xue Li, Guyu Niu, Linzhu Ren

**Affiliations:** Key Lab for Zoonoses Research, College of Animal Sciences Ministry of Education, Jilin University, 5333 Xi’an Road, Changchun 130062, China; sichen20@mails.jlu.edu.cn (S.C.); zhangliy@jlu.edu.cn (L.Z.); Lix9916@mails.jlu.edu.cn (X.L.); niugy9916@mails.jlu.edu.cn (G.N.)

**Keywords:** porcine circovirus 3 (PCV3), epidemiology, pathobiology, disease, infection

## Abstract

The recently discovered porcine circovirus 3 (PCV3) belongs to the *Circovirus* genus of the *Circoviridae* family together with the other three PCVs, PCV1, PCV2, and PCV4. As reported, PCV3 can infect pig, wild boar, and several other intermediate hosts, resulting in single or multiple infections in the affected animal. The PCV3 infection can lead to respiratory diseases, digestive disorders, reproductive disorders, multisystemic inflammation, and immune responses. Up to now, PCV3 infection, as well as the disease caused by PCV3, has been reported in many swine farms worldwide with high positive rates, which indicates that the virus may be another important pathogen in the swine industry. Therefore, we reviewed the current progress on epidemiology and pathobiology of PCV3, which may provide the latest knowledge of the virus and PCV3-related diseases.

## 1. Introduction

Porcine circovirus 3 (PCV3) belongs to the *Circovirus* genus of the *Circoviridae* family [1,2]. The genome of PCV3 is a single-stranded circular DNA of 2000 nucleotides (nt), which contains at least three open reading frames (ORFs) [3,4]. The first two ORFs, ORF1 and ORF2, respectively, encode two major viral proteins, replicase (Rep) and Capsid (Cap), while the function of the ORF3 is still unknown. Sequence analysis showed that PCV3 has low homology with that of the PCV1, PCV2, and PCV4, while the PCV3 genome is conserved and has high sequence homology among different PCV3 strains [1,2]. We previously also found that most of the PCV3 strains can be divided into two clades, clade I and clade II, using five different phylogenetic methods [2], which has been further confirmed by the recent results of other groups [5,6]. These results suggest that PCV3 genotypes based on complete coding sequences of PCV3 are stable and reliable, and the viral genome is relatively conserved compared with that of PCV2.

The virus was firstly identified in American domestic pigs with porcine dermatitis and nephropathy syndrome (PDNS)-like clinical signs, cardiac and multisystemic inflammation in 2016 [3,4]. Thereafter, the virus has also been reported in several other countries, including China, Thailand, Germany, Brazil, Denmark, Italy, India, and Spain, etc. [1,2,7,8,9,10,11,12,13,14], indicating that the virus spreads worldwide. Numerous groups reported that PCV3 infection can lead to mild to severe diseases in pig herds [13,15,16,17,18,19]. Furthermore, it has been reported that PCV3 exists in animals under different clinical conditions, including pigs, wild boar, and other animals. Since the discovery of PCV3, the literature about PCV3 has gradually increased. Although some literature reported that PCV3 has little correlation with the disease [20], recent studies on epidemiology and pathology of PCV3 have further demonstrated that PCV3 infection is related to the respiratory, digestive and/or reproductive disorders, and multisystemic inflammation [13,14]. Wang et al. also reported that co-infection of PCV2 and PCV3 leads to more serious clinical symptoms in some farms [21]. In this review, we discuss the recent progress on epidemiology and pathobiology of PCV3, which may provide updated knowledge of the virus and PCV3-related diseases.

## 2. Epidemiology of PCV3

### 2.1. Host Spectrum of PCV3

Although PCV3 was firstly discovered in the United States in 2016, retrospective studies have shown that PCV3 is closely related to bat circovirus [1,4,22,23,24], and PCV3 was also detected in clinical samples in 1950 or even earlier [25,26,27,28,29]. Codon index analysis showed that *Rhinolophus ferrumequinum* has a deeper influence on PCV3 coding sequence, followed by *Sus scrofa*, *Canis familiaris*, and *Homo sapiens* [24]. Therefore, it is assumed that PCV3 may be an ancient virus, which evolved from bats and then gradually adapted to pigs and other animals [1,22], suggesting the virus can spread across species and has a wider host spectrum.

As reported, PCV3 can infect pigs of almost all ages, including sick- and healthy-weaned pigs, sows, and pigs in slaughterhouses [11,26,30,31,32,33]. Qi et al. found that the positive rates of PCV3 in samples collected from pigs with respiratory system diseases and digestive system diseases were 26.6% and 10.4%, respectively [34]. Furthermore, PCV3 positive rates of weaned piglets with severe respiratory diseases (63.75%) or diarrhea (17.14%) were significantly higher than that of weaned piglets with mild respiratory diseases, nondiarrheal or asymptomatic piglets [31]. Moreover, the positive rate of PCV3 in healthy pigs was 6.7% (4 out of 60) [20].

Apart from domestic pigs, PCV3 has also been detected in other domestic animals, wild animals, and ticks, including wild boar, dogs, cattle, mice, etc. [7,30,35,36,37,38,39,40,41,42]. It was found that seven of 70 serum samples collected from free-living wild boars in Paraná State, Brazil were confirmed to be PCV3-specific by PCR [41]. Franzo et al. reported that one chamois (12.5%), two roe deer (4%) and 13 wild boars (44.8%) of 109 animals samples, and two ticks of *Ixodes ricinus* species (4.25%) were PCV3 positive, with an overall high positive rate among wild boar compared to that of the other wild animals [40]. Other groups reported that the positive rate of PCV3 in wild boars was 30–50% or even higher than that of the domestic pigs [1,7,13,42]. PCV3 was detected in 28.95% (434/1499) samples collected from clinically healthy cattle from 2011 to 2018 [38]. Notably, PCV3 rescued from an infectious clone can infect 6-week-old Kunming mice, resulting in significant changes in lung and heart tissues compared with that of the uninfected mice [43]. Moreover, we previously found that PCV2 can infect human cells, including normal cells and cancer cells [44], and since PCV2 and PCV3 are in the same genus, we suggest that PCV3 may infect human beings, which needs to be further evaluated [1]. These results indicate that PCV3 has a wide host spectrum, which may pose a great threat to the domestic pig and pig industry.

### 2.2. Transmission Routes

It was reported that PCV3 showed a broad histotropism, which has been detected in almost all kinds of pig tissues and fluids, including heart, liver, spleen, lung, kidney, brain, lymph nodes, tonsil, serum, feces, and oral fluids [1,15,43,45]. Among the tissues, the heart, lung, and lymph nodes contain more viral genomes than other organs [15,46]. Compared with serum (9.7%) or feces (15.0%), PCV3 is more easily detected in oral fluids (37.3%), which ranged from 10^2.5^ to 10^7.2^ copies/mL [45]. Notably, most of the infected animals, including domestic pigs and other animals, exhibit mild or asymptomatic symptoms [7,35,36,37,38,39,40,41], which may serve as a reservoir and intermediates for the virus transmission.

Furthermore, PCV3 was also detected in farrowed sows and colostrum, with a 44.74% positive rate of PCV3 in colostrum samples [18,47]. The presence of PCV3 in colostrum may be related to the presence of PCV3 in the serum of sow, as the high-viremic sows exhibited significantly higher PCV3 positive colostrum (100%), and the titer of PCV3 ranged from 4.01 to 7.33 log copies/mL [47]. A recent report showed that PCV3 was identified in the placenta of gilt and precolostrum sera of its piglets [48]. The viral load in the piglets gradually decreased from 10^6^ (in the precolostrum sera) to 10^3^ (4.93 log genomic copies/mL, in the colostrum sera) within six weeks [48]. Moreover, PCV3 has also been identified in semen samples (8.5%) by Ku et al. [49]. In addition, Wang et al. found that the positive rates of PCV3 in oral fluids and fetuses were 56.9% and 57.1%, respectively [50]. The results indicate that PCV3 can be horizontally transmitted between different hosts or intermediates, and can also be transmitted vertically through the semen, placenta, and colostrum.

### 2.3. Co-Infection

Mixed infection or co-infection of different pathogens is widespread in pig farms, which makes the diseases more complicated. Co-infection can aggravate the illness of infected animals and may also be beneficial to the proliferation of pathogens. As reported previously, co-infection of porcine reproductive and respiratory syndrome virus (PRRSV), porcine epidemic diarrhea virus (PEDV), porcine parvovirus (PPV), swine influenza A virus (swIAV), classical swine fever virus (CSFV), or torque teno sus virus (TTSuV) with PCV2 was detected worldwide [51]. Especially, the double, triple or even multiple co-infections of PCV2 with other pathogens are widespread in pigs, among which the triple, double and quadruple co-infection accounts for the majority [1,51]. As PCV3 belongs to the same genus of PCV2, it is speculated that PCV3 infection may increase co-infection with other causative pathogens of swine diseases. A recent report from Serbia showed that PCV3 was detected in pigs suffering from PRRSV infection, *Actinobacillus pleuropneumonia* infection, *pneumonic* *pasteurellosis* infection, Glässer’s disease, or porcine respiratory disease complex (PRDC) [52]. Therefore, the confirmation and investigation of PCV3 co-infection with other pathogens should also be one of the focuses of current research.

Among the swine pathogens, co-infection of PCV2 and PCV3 was the most reported in swine farms [21,50,51,53,54,55,56,57,58]. Xia and colleagues found that the infection rates of PCV2, PCV3, and PCV2-PCV3 co-infection were 50.0%, 13.3%, and 6.78%, respectively, in China from 2015 to 2018 [56]. Wang et al. reported that the positive rate of PCV3 in 169 tissue samples collected from Tianjin, China from 2016 to 2018 was 37.3%, while the co-infection rate of PCV2 and PCV3 was 14.8% [21]. Furthermore, the co-infection rate of PCV2 and PCV3 was 27.6% in 340 clinical samples collected from the diseased pigs of 15 farms in Henan province in the first half of 2017 [55]. Xu et al. found that the positive rates of PCV2, PCV3, and PCV2-PCV3 co-infection accounts for 57.07% (113/198), 36.36% (72/198), and 19.7% (39/198), respectively, in 198 samples collected from 2018 to 2020 in China [54]. Moreover, Wang et al. evaluated 2125 porcine samples collected from 910 cases in the Midwest of the USA between 2016 and 2018 and found that the co-infection rate of PCV2 and PCV3 gradually increased from 3.4% in 2016 to 16.1% in 2018 [50]. Notably, a survey of 624 serum samples from clinically healthy pigs of nine European countries, including Spain, Belgium, France, Germany, Italy, Denmark, Netherland, Ireland, and Sweden, showed that positive rates of PCV2, PCV3, and PCV2-PCV3 co-infection were 21%, 8%, and 3% in sera from fattening pig, respectively [53]. In addition, a report from South Korea showed that positive rates of PCV2, PCV3, and PCV2-PCV3 co-infection were 21.7%, 6.5%, and 28.3%, respectively [57]. These results suggest that the prevalence of PCV2 and PCV3 co-infection is widespread all over the world and has gradually increased in recent years.

Other porcine pathogens, especially viruses, co-infected with PCV3 were also found in healthy or sick pigs, such as PEDV, PRRSV, PCV4, pseudorabies virus (PRV), CSFV, and PPV, etc. [59,60,61,62,63,64,65,66,67,68,69]. PEDV is another common virus reported to be co-infected with PCV3 [59,60,61]. Guo et al. found that most of the 76 enteric samples collected from suckling piglets with severe diarrhea were mixed infection, and the co-infection rates of PCV2/PCV3, PCV2/PEDV, PCV3/PEDV were 69.74%, 57.89%, and 53.95%, respectively [59]. Geng et al. revealed that the PCV3 positive rate accounted for 67.1% of 283 clinical samples collected from 2014 to 2017 in pig herds of Zhejiang province, China [60]. The single infection rate of PCV3 was 30%, while the co-infection was the majority, mainly PEDV (41.6%) [60]. Han et al. reported that from 2014 to 2018, the single infection rates of PEDV and PCV3 accounted for 43.94% (29/66) and 16.67% (11/66), respectively, while the co-infection rate of the two viruses was 27.27% in 66 intestinal and fecal samples from piglets suffering from diarrhea at different pig farms in Hebei and Henan provinces, China [61]. Moreover, other viruses, such as PRRSV, TTSuV, PCV4, pseudorabies virus (PRV), CSFV, and PPV, have also been reported to be co-infected with PCV3 [62,63,64,65,66,67,68,69]. The co-infection rates of PCV3 with CSFV, PRV, PCV4, PRRSV, PPV6, and PPV7 were 6.92%,14.53%,17.19%, 36.36%, 60%, and 74.2% in some pig farms in China, respectively [63,66,67,68,69]. These results indicate that the co-infection of PCV3 with other pathogens is a common condition in pig farms in China.

Apart from double infection, triple and/or multiple infections of PCV3 with other viruses were also detected in clinical samples. As reported by Zheng et al., TTSuV1 and TTSuV2 were detected in 110 of 132 (83.3%) and 94 of 132 (71.2%) of PCV3-positive samples, while 66 of 132 (50.0%) PCV3-positive samples were co-infected with both TTSuV1 and TTSuV2 [65]. Furthermore, the triple infection rate of PCV2, PCV3, and PEDV was even up to 48.68% in some pig farms [59]. Chen et al. reported that triple infections of CSFV + PRRSV + PCV2 and PRRSV + PCV2 + PCV3 were 2.52% and 0.63%, respectively, in 159 pigs collected from 63 herds of eight Chinese regions during 2016–2018 [64].

It is worth noting that the co-infections of PCV3 with PCV2 and/or PPV were common in both domestic and wild boars [70]. Dei et al. evaluated blood and tissue samples from 189 *Sardinian suids* (34 domestic pigs, 115 feral free-ranging pigs, and 39 wild boars) and found that the infection rates of PCV3 in the three groups were 17.64%, 77.39%, and 61.54%, respectively [70]. Moreover, whether co-infection is related to more severe clinical symptoms needs to be further clarified. As Wang et al. reported, co-infection of PCV2 and PCV3 leads to more serious clinical symptoms in some farms [21].

These results indicate that it is necessary to further systematically evaluate and investigate the pathogenicity and multiple infections of PCV3 and other porcine pathogens and to develop an effective vaccine against PCV3 as well as the co-infections.

## 3. Pathobiology of PCV3

### 3.1. Respiratory Diseases or Digestive Disorders

As reported, PCV3 infection may be closely related to porcine respiratory disease or digestive disorders, which are the most common clinical symptoms in pigs, especially in weaned litters [3,31,34]. Zhai et al. reported that the PCV3-positive rate of weaned piglets with severe respiratory disease (SRD) was significantly higher (63.75%, 51/80) than that of weaned piglets with mild respiratory disease (MRD) (13.14%, 23/175) and asymptomatic piglets (1.85%, 4/216). Meanwhile, the positive rate of PCV3 in weaned piglets with diarrhea was significantly higher (17.14%, 6/35) than that in non-diarrheal weaned pigs (2.86%, 1/35) [31]. According to another survey by Qi et al., the positive rates of PCV3 in samples affected by digestive diseases or respiratory diseases were 10.4% (50/480) and 26.6% (25/94), respectively [34]. In addition, compared with asymptomatic pigs, more viral loads were detected in weaned or fattened pigs with obvious symptoms [31]. Moreover, Jiang et al. infected 6-week-old Kunming (KM) mice with the rescued PCV3 virus and found that there was no significant difference in tissues/organs between the control group and the infected group [43]. However, alveolar epithelial cells in the infected group proliferated in the local area of the lung and became congested at the local lobule edge [43].

The histopathological features of pigs with intestinal symptoms include atrophic villi and crypt, and changes in gut microbiota [71,72]. Zhang et al. found that 27.28% and 14.29% of PCV3-positive suckling and weaned piglets were associated with diarrhea, respectively [71]. The villi of the duodenum, jejunum, and ileum atrophied moderately to severely, and the average height of villi and depth of crypt decreased significantly [71]. Hou and his colleagues evaluated the dynamic composition of gut microbiota following PCV3 infection and found that diversity and richness of gut bacteria in the PCV3-infected group were different from those of the mock group, with an obvious decrease of *Clostridium_sensu_stricto_1* and significant difference of functional bacteria in PCV3-infected pigs compared with that of the mock-infected pigs [72]. Moreover, mucosal epithelial cells and lymphocytes of the PCV3-infected group were partially necrotic, with a large number of eosinophils, lymphocytes, and a small number of plasma cells infiltrated [72]. PCV3 antigen-positive cells were also detected in the small intestine of a PCV3-infected piglet [72].

### 3.2. Multisystemic Inflammation and Immune Responses

PCV3 was first detected in pigs with cardiac and multisystemic inflammation [4]. Thereafter, numerous pieces of evidence proved that PCV3 is associated with multiorgan lymphoplasmacytic periarteritis, lymphocytic myocarditis, lymphoplasmacytic meningoencephalitis, and/or PDNS [16,73,74]. Alomar et al. reported the investigation of PCV3-infected pigs and found that there were no obvious gross lesions in viscera, but multiorganic moderate to severe lymphoplasmacytic periarteritis and arteritis were observed in artery, heart, kidney, spleen, portal arteriole, meninges, lung, and/or stomach, characterized by inflammatory infiltration of lymphoplasmacytes [16]. Furthermore, the connective tissue and adventitia around the artery intima were destroyed by inflammatory infiltration, smooth muscle cells showed mild to severe vacuolation and loss of cytoplasmic boundary, endothelial cells expanded into the lumen, and leukocytes adhered to the endothelium [16]. Moreover, mild meningoencephalitis, myocarditis, interstitial pneumonia, nephritis, periportal hepatitis, rhinitis, and periarteritis characterized by lymphoplasmacytic infiltration were observed in several sick pigs [16]. These results suggest that lymphoplasmacytic infiltration is the main feature of multisystemic inflammation in PCV3-infected pigs.

Apart from multisystemic inflammation, viremia, IgG response, and viral shedding were obviously prolonged in PCV3-affected animals [73,74,75]. Temeeyasen et al. revealed the immunopathogenesis of PCV3 in cesarean-derived, colostrum-deprived (CD/CD) pigs [73]. As result, viremia was detected in the PCV3-infected group from 3 days post-inoculation (dpi) to the end of the study, and nasal shedding was detected from 3 to 28 dpi [73]. Moreover, PCV3 infection-induced IgG response, which first appeared on 7 dpi and lasted until the 42nd dpi, but there was no significant T-cell response [73]. Mora-Díaz et al. found that viremia can be detected from 7 to 28 dpi in all PCV3-inoculated CD/CD pigs, and 5 of 8 PCV3-inoculated pigs showed IgM positive from 7 to 14 dpi [74]. In addition, the levels of proinflammatory cytokines and chemokines in piglets inoculated with PCV3 were significantly increased, including interleukin 1 beta (IL-1β), IL-6, IL-23α, interferon-gamma (IFN-γ), tumor necrosis factor-alpha (TNF-α), and chemokine ligand 5 (CCL5) [75]. A recent study showed that PCV3 capsid protein (Cap) stimulated the activation of NF-κB and upregulated pro-inflammatory cytokine expression, including IL-6 and TNF-α, via RIG-like receptor (RLR) and Toll-like receptor (TLR) signaling pathways in vitro [76]. However, the viral Cap significantly inhibited IFN-β-stimulated response element (ISRE) activity by interacting with the transactivation domain of signal transducer and activator of transcription (STAT2) [77]. Zhang et al. found that PCV3 Cap can also interact with GTPase-activating protein-(SH3 domain)-binding protein 1 (G3BP1), and thus preventing the binding between cyclic GMP-AMP (cGAMP) synthase (cGAS) and interferon-stimulating DNA (ISD), resulting in an inhibition type I interferon induction [78]. Therefore, it was supposed that PCV3 inhibits IFN signaling and activates NF-κB signaling to modulate host innate immunity and inflammatory responses. However, the exact mechanism and pathogenesis of PCV3 remain to be elucidated. Moreover, it is well known that PCV2 infection may lead to immunosuppression, followed by secondary infection of other pathogens [51,79], whether PCV3 can cause immunosuppression needs further clarification.

### 3.3. Reproductive Disorders

PCV3 can be detected in the gilt of the pro-farrowing and farrowing period, in serum or tissues of weak-born piglets, in mummified fetuses and placenta, in the precolostrum sera of piglets and colostrum, suggesting the virus is associated with the reproductive disorders [33,48,74,80,81,82]. Among the collected samples, the highest load of the PCV3 genome was detected in the thymus and lymph nodes of aborted and weak-born piglets [83]. Although PCV3 can infect sick and healthy pigs, however, Zou et al. found that the positive rate of PCV3 in sows with reproductive disorders was significantly higher than that of the healthy sows (45.9% vs. 21.9%) [32]. These results indicated that PCV3 might infect and replicate in sow and piglets, resulting in vertical transmission and reproductive failure.

Furthermore, Dal Santo et al. found that 270 of 276 mummified fetuses collected from 11 commercial swine farms in Brazil were PCV3-positive [80]. Serena et al. from Argentina evaluated mummified and stillborn piglets from cases of chronic reproductive disorder and normal deliveries and found that 100% PPV-positive mummified and/or stillborn piglets were PCV3-positive [82]. Of the 131 fetuses collected from three different sows, three were positive for PCV3, but negative for PPV, PCV2, ADV, *Leptospira* spp., and *Brucella* spp. [82]. The percentage of mummies (7% vs. 3.2%) and stillborn pigs (7.2% vs. 4.2%) were increased, while the total number of live pigs decreased (86% vs. 92%) in PCV3-affected farms compared with that of the reference farms [82]. Saporiti et al. evaluated the positive rate of PCV3 in primiparous and multiparous sows as well as their respective fetuses [33]. The results showed that sera of multiparous sows were negative, whereas 33.3% of primiparous sows were positive for PCV3 [33]. In total, 86 of 255 fetuses were PCV3 positive, and the detection rate of PCV3 in fetuses from primiparous sows was significantly higher than those of the multiparous derived fetuses [33]. These results indicate that PCV3 might cause intrauterine infection, resulting in mummies and stillborn piglets.

Moreover, co-infections of PCV3 with other pathogens may also lead to reproductive failure or even increase the severity of the disease [80,82,84]. As reported by Dal Santo et al., 93.1% of 270 PCV3-positive mummified fetuses were co-infected with PPV, PCV2, and/or *Leptospira* spp. [80]. Mai et al. also found that the positive rates of PPV7 in PCV3-positive reproductive failure-serum and PCV3-positive aborted fetus samples were significantly higher than that of the PCV3-positive non-reproductive failure serum samples [84]. Meanwhile, the positive rates of PCV3 in PPV7-positive samples were obviously higher than that of the PPV7-negative samples [84]. These results further confirmed that the co-infection of PCV3 with other pathogens may enhance the infection of PCV3, and vice versa, resulting in more severe diseases.

## 4. Conclusions and Perspective

PCV3 infection may be closely related to reproductive disorders, respiratory disease, and multiorganic inflammation. Similar to PCV2, which has caused great harm to the swine industry, PCV3 infection occurs in pigs and wild boars as well as numerous intermediate hosts, resulting in horizontal and vertical transmissions. Notably, it was reported that the virus can infect multiparous sows and live-born piglets, showing obvious clinical symptoms or subclinical symptoms [85], suggesting the virus also has potential harm to the swine industry. However, except for previous studies using infectious clones [43,75], PCV3 infection in CD/CD pigs showed that lesions and clinical signs were mild and limited in PCV3-infected CD/CD pigs [73,74]. Moreover, Saporiti et al. reported that there is a similar percentage of PCV-3 in the serum of sick pigs and healthy pigs, indicating that this virus seems to have no causal relationship with respiratory or intestinal diseases [20], which is controversial with other reports. Therefore, we should continue to closely monitor the prevalence and co-infection of PCV3 with other swine pathogens, as well as the co-factors of the disease manifestation. The dynamic changes of genetic diversity and molecular epidemiology of PCV3 dominant strains should be continuously tracked. Furthermore, Oh and colleagues recently have demonstrated successful isolation of PCV3 using primary porcine kidney cells [86]. However, the limited culture cells of the virus limit the in vitro propagation and research of the virus. Therefore, PCV3 rescued by reverse genetics [43,75] may provide useful information for studying the pathogenic mechanism of PCV3 and preventing and controlling the disease. Meanwhile, it is important and urgent to analyze the conserved epitopes of currently identified PCVs (PCV1, PCV2, PCV3, and PCV4) or develop universal antibodies and/or antiviral strategies against PCVs that will appear or reappear in the future.

## Data Availability

All data generated or analyzed during this study are included in this published article.
